# Bio-based nanophotothermal systems as smart drug delivery platforms for multimodal synergistic cancer therapy—a review

**DOI:** 10.3389/fmedt.2026.1943029

**Published:** 2026-09-28

**Authors:** Aleksandra Porjazoska Kujundziski

**Affiliations:** Faculty of Engineering, International Balkan University, Skopje, North Macedonia

**Keywords:** bio-based systems, drug delivery, cancer treatment, chitosan, hyaluronic acid, nanophotothermal systems, theranostics

## Abstract

Conventional protocols for cancer treatment are usually accompanied by serious limitations, which can be addressed using innovative, minimally invasive methods, such as photothermal therapy (PTT). But, using this method as a standalone therapy is usually accompanied by uneven heat distribution within tumor tissues, and the risk of cancer recurrence. Integrating PTT with complementary therapeutic modalities can help prevent these issues. The development of nanophotothermal systems (NPTSs) as multipurpose, multimodal platforms for smart drug delivery has greatly broadened the therapeutic landscape of cancer treatment. Additionally, recent developments in bio-based and biocompatible nanomaterials have further expanded the potential of NPTSs in clinical applications. Furthermore, NPTSs combine therapeutic functionalities and diagnostic imaging, i.e., tracking modalities, such as photoacoustic, magnetic resonance, or fluorescence imaging, to provide real-time monitoring of drug delivery and treatment response. Translation of preclinical trials into clinical applications for these systems remains challenging due to issues such as toxicity, biodegradability, scalability, standardization, and regulatory approval. This narrative review analyzes two of the most versatile natural polysaccharides, chitosan (CS) and hyaluronic acid (HA), which have complementary physicochemical and biological properties. Covering the advantages of CS and HA for the application in multifuntional and multimodal nanophotothermal systems, their complementary effects, as well as their functional implications in therapeutic settings, this work aims to create a framework that will serve as a basis for further research in optimizing the design of multifunctional drug-delivery nanosystems, and at the same time, facilitate the transition of photothermal therapy from the bench to clinical use.

## 1 Introduction

Regardless of the remarkable advances in biomedical research and clinical oncology, and progress made in molecular diagnostics, imaging technologies, and precision medicine, which greatly improved the understanding of tumor biology, effective treatment for many cancers remains unavailable, so that cancer remains one of the leading causes of morbidity and mortality worldwide. Recent estimations indicate millions of newly diagnosed cases of cancer and deaths annually, and in Europe alone, approximately one-quarter of all deaths in 2023 were attributed to cancer (1).

Many conventional strategies for cancer treatment, including surgery, chemotherapy (CT), radiotherapy (RT), hormone therapy, targeted therapy, and, more recently, immunotherapy, remain associated with significant clinical problems, such as limited tissue selectivity, heterogeneous patient responses, diverse mechanisms of tumor resistance, rapid cellular adaptation, and others. The consequences are often cancer recurrence and metastasis (2, 3).

Nanotechnology and nanoscale drug delivery systems (DDSs) overcome many limitations of conventional treatments, including improved systemic drug solubility, protection of bioactive molecules from premature degradation, enhanced accumulation in tumors via passive and active targeting, controlled drug release, and others (4, 5). Therefore, these attributes have positioned nanomedicine and nanoplatforms as multifunctional systems that integrate therapeutic, diagnostic, and imaging capabilities into a single construct, as a promising approach for precision oncology, offering personalized treatment, improving therapeutic efficacy, and reducing systemic toxicity (3, 6).

An equally important development has been the emergence of stimuli-responsive nanoplatforms that exploit both endogenous and exogenous triggers to enable controlled therapeutic activation (7, 8). Characteristics of the tumor microenvironment (TME)—including acidic pH, hypoxia, elevated reactive oxygen species, abnormal enzyme expression, and altered redox conditions—serve as biological triggers for selective drug release, while external stimuli such as near-infrared irradiation provide precise temporal control of photothermal activation. Together, these multi-responsive systems considerably improve treatment selectivity and reduce systemic toxicity (9, 10).

Photothermal therapy (PTT) has attracted considerable attention in cancer treatment due to its minimally invasive effect for selective tumor ablation (6, 11, 12). Employing photothermal agents (PTAs) that, upon absorbing near-infrared (NIR) light, enable the PTT approach to make irreversible damage to malignant cells while minimizing adverse effects on surrounding healthy tissues (7, 13, 14). Localized hyperthermia not only destroys tumors but also provides other benefits during PTT therapy, such as enhancing vascular permeability, facilitating drug penetration, accelerating intracellular drug release, sensitizing tumors to conventional therapy, and promoting immunogenic cell death (11, 15).

Despite many advantages, the literature indicates that when used alone, PTT rarely achieves complete tumor eradication. Limited penetration of light into deep tissues, heterogeneous heat distribution within tumors, incomplete tumor ablation, and the induction of heat shock proteins that promote tumor survival contribute to the limited effectiveness of PTT. An additional problem occurs from potential residual malignant cells that survive suboptimal thermal treatment, which may contribute to tumor recurrence and the development of distant metastases. An efficient solution to these and similar issues is the application of integrated multimodal therapeutic strategies that combine photothermal monotherapy with complementary treatment modalities to exploit synergistic mechanisms (8, 16, 17).

Many studies have reported remarkable progress in nanophotothermal systems (NPTSs), which, beyond simple targeting agents, integrate CT, photodynamic therapy (PDT), radiotherapy (RT), gene therapy (GT), and immunotherapy (IO) and have evolved into intelligent, multifunctional drug-delivery platforms. These complementary multiple mechanisms significantly improve therapeutic efficacy while reducing the doses required for individual therapeutic modalities, making multimodal synergistic therapy one of the most promising directions in contemporary precision oncology (8, 15, 16, 18).

Another important feature of modern nanophotothermal systems (NPTSs) is the integration of therapeutic and diagnostic functions within a single nanoplatform, i.e., the development of theranostic systems. Such systems combine targeted drug delivery with real-time monitoring via imaging modalities (e.g., photoacoustic or magnetic resonance imaging), enabling clinicians to plan treatment and, during the treatment, to monitor nanoparticle accumulation, evaluate therapeutic response, and optimize therapeutic protocols. These capabilities are expected to play an increasingly important role in precision oncology by enabling individualized treatment strategies (2, 7).

Photothermal materials of inorganic origin demonstrated superior photothermal conversion efficiency and efficacy in cancer ablation, but they also face issues with long-term biocompatibility and persistence in the body, limited biodegradability, and potential toxicity, which hinder their translation into clinical settings (9, 19). Simultaneously, their biodegradable alternatives, such as the naturally derived polymers, attracted increasing attention. In addition to their biocompatibility, bio-based polymers (20–26) offer excellent immunogenicity, presence of functional groups for chemical modification, and intrinsic responsiveness to physiological stimuli. These properties make bio-based materials particularly attractive for constructing multifunctional nanoplatforms with improved biosafety and translational potential.

Although remarkable progress has been made, several challenges, namely, the lack of large-scale manufacturing and scalability, unclear pharmacokinetics and pharmacodynamics, biodistribution, long-term biosafety, regulatory approval, and standardization, continue to impede the clinical translation of multifunctional nanophotothermal platforms. Therefore, the development of clinically applicable nanomedicine platforms requires close collaboration among different specialists and regulatory agencies (10).

The conceptual design of this review, illustrating the evolution from conventional cancer therapies to bio-based nanophotothermal DDSs designed for multimodal synergistic cancer therapy, is presented in Figure 1.

**Figure 1:**
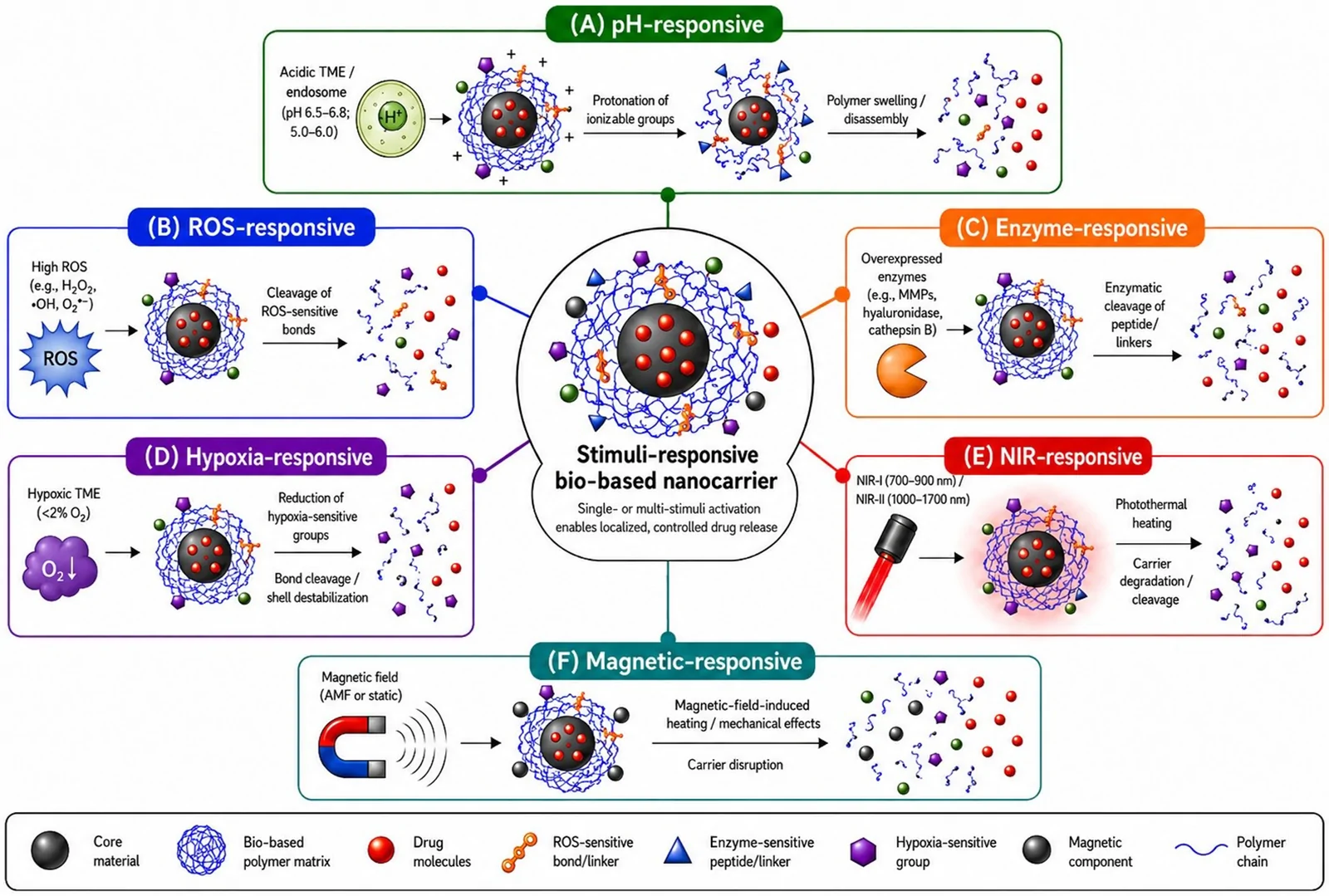
Conceptual evolution from conventional cancer therapy to smart bio-based nanophotothermal platforms for multimodal synergistic cancer therapy. **(A)** Conventional cancer treatments are associated with limitations including non-specific toxicity, drug resistance, limited tumor selectivity, and recurrence, motivating the development of safer and more selective approaches. **(B)** Utilizing various nanocarriers, drug encapsulation, solubility in body fluids, cargo protection from earlier decomposition, circulation, and tumor delivery are improved. **(C)** Irradiated by NIR-I/NIR-II, photothermal agents convert absorbed photon energy into heat, resulting in localized hyperthermia and tumor-cell damage/ablation. **(D)** Bio-based nanophotothermal systems (NPTSs) as biocompatible, biodegradable, biomimetic, and functionalizable components can serve as carriers, coatings, stabilizers, targeting interfaces, and/or photothermal matrices. **(E)** Integration of NPTAs in smart drug delivery systems enables various functionalities: high drug loading, stimuli-responsive and NIR-triggered release, active targeting, imaging guidance, controlled release, and theranostics. **(F)** Integration of PTT with chemotherapy, chemodynamic therapy (CDT), immunotherapy, radiotherapy, and gene therapy within a single platform—multimodal synergistic treatment—enhanced therapeutic efficacy, reduced systemic toxicity, and tumor recurrence. The figure was generated using GPT 5.6 Sol model (27) and subsequently reviewed and refined for scientific accuracy and content.Source: OpenAI ChatGPT (GPT-5.6 Sol).

Unlike previous reviews that primarily classify bio-based nanomaterials by material type, this study adopts a structure–function perspective by comparing two complementary design philosophies, represented by CS and HA. With an analysis of recent advances in material design, functional engineering, therapeutic performance, and the translational challenges of these systems, this article attempts to provide a comprehensive framework for the future development of safe, biodegradable, and clinically relevant nanophotothermal platforms to advance precision cancer therapy.

This study conducted a narrative literature review using the Web of Science and Scopus databases, focusing on open-access review and empirical research articles written in English. Using Boolean queries containing the terms bio-based DDSs, NPTAs, cancer treatment, CS, HA, and theranostics, 82 papers extracted focusing on the period between 2021 and 2026, and 5 sources from the own database were used in the following discussion.

## 2 Fundamental principles of bio-based nanophotothermal drug delivery systems

The design of bio-based nanophotothermal DDSs is a highly interdisciplinary field that strongly depends on the relationship between material structure and therapeutic function, structured around nanotechnology, light-to-heat conversion, biomimetic targeting, and stimuli-responsive drug release. To encapsulate photothermal agents (PTAs) and therapeutic payloads, these systems utilize bio-based or bio-inspired materials (28–30).

Various drug-loading mechanisms have been employed for NPTSs, including physical encapsulation, electrostatic attraction, hydrophobic interactions, hydrogen bonding, π–π stacking, host–guest complexation, and covalent conjugation, thereby determining key parameters of DDSs, including loading efficiency, colloidal stability, drug leakage, release kinetics, and biological performance. For example, π–π interactions are characteristic of the encapsulation of aromatic drugs, or photosensitizers, within aromatic systems such as polydopamine nanoparticles, graphene oxide composites, and carbon nanodots. On the other hand, liposomes, hydrogels, albumin carriers, and polysaccharide-based matrices can encapsulate both hydrophilic and hydrophobic molecules (29). In addition, these platforms can co-load chemotherapeutics, photosensitizers (31), immune adjuvants, nucleic acids, or metabolic modulators (29, 30).

A key advantage of these systems is their capacity for stimulus-responsive release, where the therapeutic payload is released only when triggered by specific internal or external stimuli, as indicated in Figure 2. In addition to the tumors’ pathological features, external stimuli can trigger selective drug release or nanoparticle disassembly. NIR irradiation provides an external stimulus that can precisely activate heating and accelerate release only when and where treatment is required. This dual responsiveness—activation of the internal tumor microenvironment plus external photothermal control—is central to smart nanophotothermal drug delivery (9, 10).

**Figure 2:**
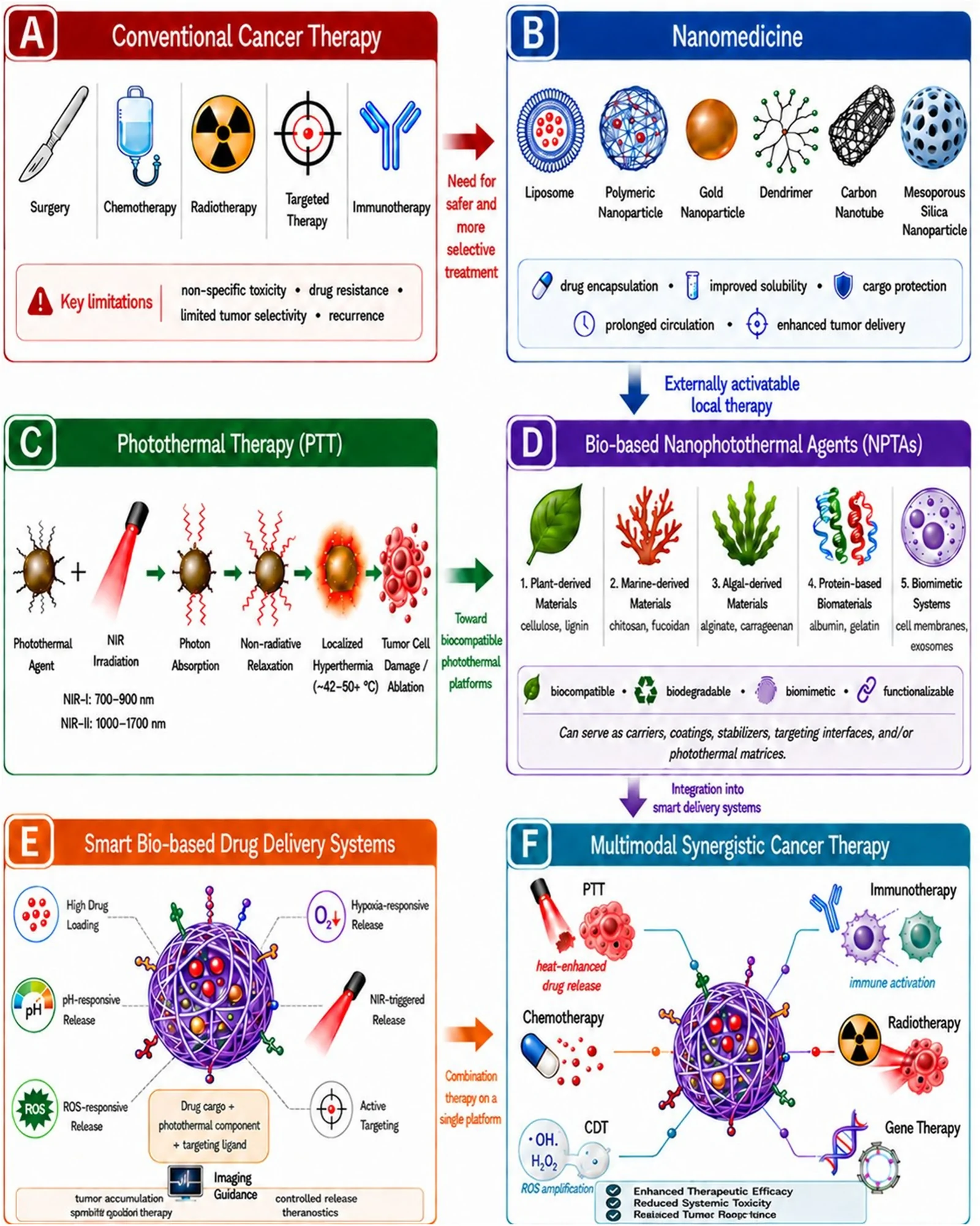
Stimuli-responsive mechanisms of controlled drug release from bio-based nanocarriers. Endogenous mechanism: **(A)** Protonation of ionizable groups by an acidic extracellular environment or endosomal conditions, followed by polymer swelling and carrier disassembly. **(B)** Reactive oxygen species (ROS)-mediated cleavage. **(C)** Cleavage of enzyme-sensitive peptides/linkers by overexpressed tumor-associated enzymes. **(D)** Under low-oxygen conditions, reduction of hypoxia-sensitive groups, leading to bond cleavage or carrier shell destabilization. Exogenous mechanisms: **(E)** NIR-I/NIR-II irradiation, producing photothermal heating and subsequent carrier degradation or cleavage. **(F)** Alternating magnetic fields induce magnetic heating and/or mechanical forces, or static magnetic fields enable magnetic manipulation, resulting in carrier disruption or controlled release. Enhanced spatiotemporal control of drug release and therapeutic activation can be provided with the individual or combined mechanisms. The figure was generated using GPT 5.6 Sol model (27) and subsequently reviewed and refined for scientific accuracy and content. Source: OpenAI ChatGPT (GPT-5.6 Sol).

Depending on their structure, nano DDSs obey passive or active tumor targeting. In passive targeting, nanosystems accumulate in tumors due to the Enhanced Permeability and Retention (EPR) effect (29, 32). In contrast, for functionalized nanosystems with ligands (e.g., folic acid, RGD peptides, antibodies), active targeting is possible when the ligands bind to receptors overexpressed on cancer cells (9, 29). Bio-based materials such as CS (25), HA (33), and others (20, 22, 23, 34) are particularly attractive in this context because they offer chemical versatility, promoting drug binding, chemical modification, ligand conjugation, hydrogel formation, and stimuli-responsive behavior, making them suitable for active targeting. Additionally, homotypic targeting occurs when nanoparticles are coated with cancer cell membranes, allowing them to selectively recognize and bind to homologous tumor tissues through inherent adhesion molecules, i.e., homotypic recognition (29).

The activation mechanism of nanophotothermal DDSs is PTT, in which PTAs absorb near-infrared light and convert light into localized heat. Raising the local temperature to 41–46 °C, causing hyperthermia, induces heat-shock damage and promotes tumor apoptosis. On the other hand, temperatures above 48–50 °C, resulting from protein denaturation and membrane disruption, cause rapid necrosis (14). The preferred NIR range is between 700 and 1350 nm because it penetrates biological tissues deeply with minimal absorption or scattering by water and hemoglobin (12, 14). The therapeutic value of bio-based NPTSs is most evident in multimodal synergistic therapy. These entities rarely act alone. They are designed to amplify the effects of other modalities, such as chemo, immuno-PTT, or gene therapies (11). As far as the chemo-PTT synergistic effect is concerned, PTT increases cell membrane permeability and vascular perfusion, thereby facilitating deeper penetration and enhanced uptake of chemotherapeutic drugs (16) such as doxorubicin (DOX) (11, 12).

At the same time, many bio-based NPTSs are hybrid by design, meaning that the biocompatibility, biodegradability, and functional adaptability of natural polymers or biomimetic coatings are often combined with the high photothermal conversion efficiency and imaging potential of inorganic or organic photothermal cores such as gold nanostructures, graphene derivatives (7, 35), carbon dots (22), semiconducting polymers (10), iron oxides, tantalum-based particles (36), or other photoresponsive materials (2), into a single therapeutic architecture. Such examples include gold–graphene hybrids (7), plant-synthesized gold nanotheranostics (37), polymerized carbon-coated metal nanoparticles (38), and photoresponsive inorganic nanomaterials (2).

Modern NPTSs usually integrate theranostic functions, combining drug delivery, stimulus response, and cancer therapy with imaging modalities such as fluorescence, photoacoustic, magnetic resonance, and others, thereby enabling monitoring of nanoparticle accumulation and irradiation parameters and assessment of treatment response, Figure 3. Several systems that combine therapeutic action with diagnostic feedback have been reported in the literature, including carbon nanodots (31), tantalum-based nanoparticles (36), gold nanomaterials (19), graphene-based hybrids (7), liposomal photoresponsive carriers (8, 39), and biomimetic membrane-coated systems (29). Such theranostic capability is important for cancer treatments because it supports more individualized treatment planning and is essential for future clinical translation.

**Figure 3:**
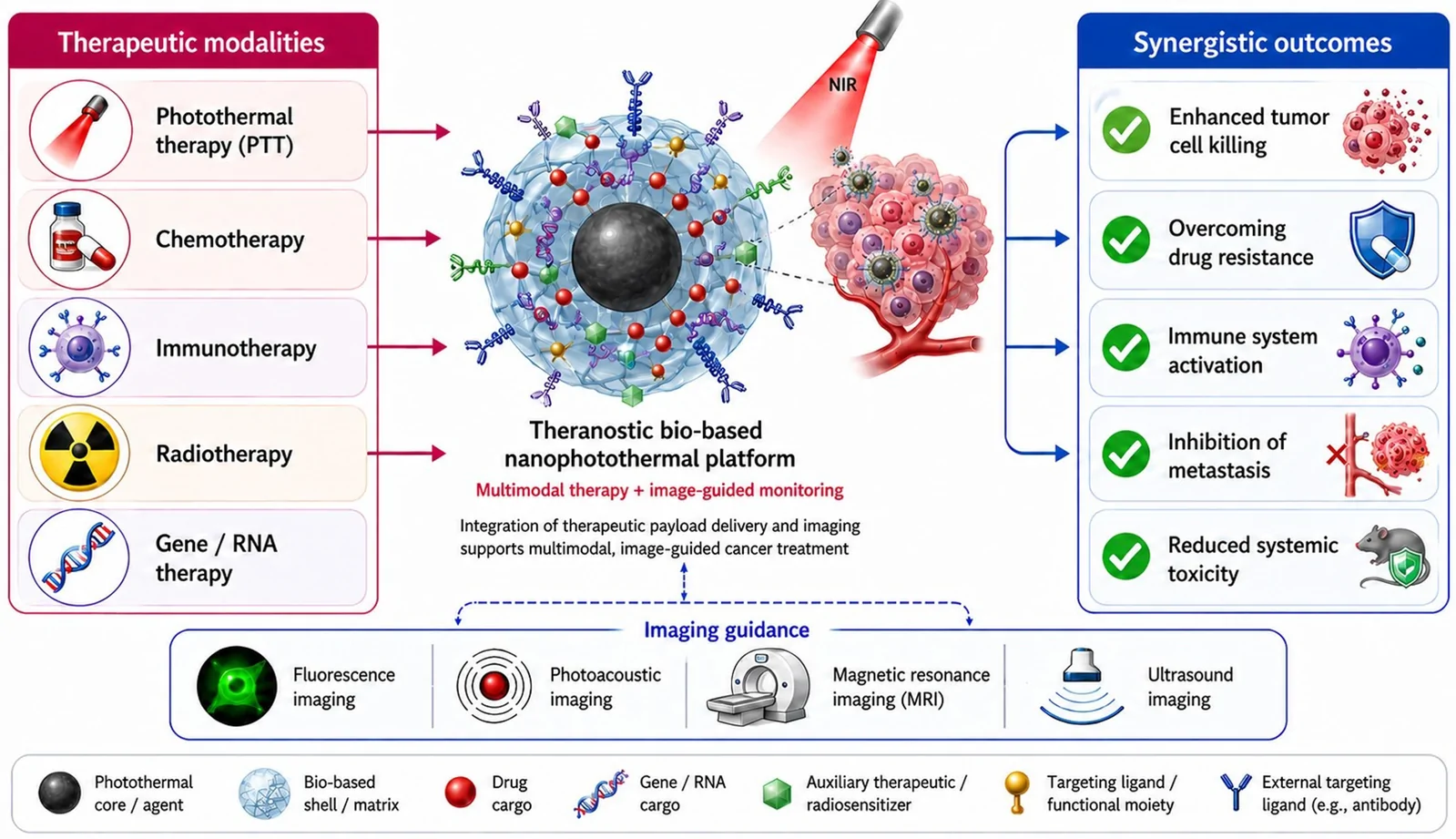
Multi-functional bio-based nanophotothermal platforms for multimodal synergistic cancer therapy integrating theranostic functions for precision cancer treatment. The figure was generated using GPT 5.6 Sol model (27) and subsequently reviewed and refined by the authors for scientific accuracy and content. Source: OpenAI ChatGPT (GPT-5.6 Sol).

All of the above indicate that bio-based nanophotothermal DDSs should be seen as integrated therapeutic platforms rather than simple nano-DDSs. Thus, scientific interest focuses on bench-to-bedside translation (3), aiming to provide controllable, safe, and reproducible systems. It is considered that the challenges in clinical translation can be addressed with the appropriate planning of the nanoparticles’ shape and sizes, ability of photothermal conversion, the methods of drug loading and release, opportunity for tumor targeting, multimodal therapies, but the basic thing to achieve all the requirements is the selection of an appropriate material. Therefore, the following part of this article will focus on the analysis of the two most common materials, CS and HA, used in the development of NPT DDSs.

## 3 Bio-based nanophotothermal platforms

Bio-based and biomimetic platforms leverage components of natural or biological origin to address the limitations of synthetic materials, such as immune recognition and poor tumor localization (29). Systems prepared from synthetic materials can be “cloaked” in membranes derived from red blood cells, cancer cells, or immune cells (e.g., neutrophils, macrophages), thereby achieving cell-membrane camouflage. This confers a “stealth” effect by bypassing the reticuloendothelial system and extending circulation time (40).

In addition to biocompatibility, bio-based materials such as chitosan (25), hyaluronic acid (33), alginate (20), cellulose (22), gelatin (40), albumin (41), silk fibroin (42), lignin (23), and others (43) are particularly attractive because of their chemical versatility, i.e., the presence of functional groups promoting chemical modification, drug binding, ligand conjugation, hydrogel formation, promoting stimuli-responsive behavior, enable these materials suitable for active targeting.

Bio-based NPTPs are often engineered as hybrid systems that combine natural or bioinspired materials with stimuli-responsive agents, such as gold nanostructures, graphene derivatives, carbon dots, iron oxides, and semiconducting polymers, integrating properties like high photothermal efficiency and imaging capability with biodegradability, functional adaptability, and improved biocompatibility. Such a design can “trick” the immune system and circulate longer in the bloodstream. Many of these materials, such as HA and certain proteins, have natural affinities for specific receptors on cancer cells, which facilitate active drug targeting (25). Furthermore, albumin prevents drugs from dissolving too quickly and prolongs their time in the bloodstream (41), and chitosan imparts pH sensitivity and mucoadhesive properties (25). Also, it was shown that alginate (20) and gelatin (25) are useful for injectable hydrogels and localized therapy, cellulose (22) and lignin (23) provide renewable structural platforms, and polydopamine combines bioinspired adhesion, strong NIR absorption, surface reactivity, and photothermal conversion (43).

Based on their origin and structural characteristics, bio-based materials employed in NPT drug delivery can be broadly classified into polysaccharides, polyphenolic and bio-inspired organic polymers, proteins and protein-based scaffolds, and biomembrane-based (biomimetic) systems. Representative materials, their principal functional characteristics, and their roles in nanophotothermal drug delivery are summarized in Table 1.

**Table 1:** Classification and functional characteristics of representative bio-based materials used in nanophotothermal drug delivery systems.

Materials class		
Material	Characteristics	Ref.
Polysaccharides (Bio-based Polymers)—natural carbohydrate polymers widely used for their stability and ability to form hydrogels		
Chitosan	Highly valued for its biocompatibility, biodegradability, and negligible toxicity, is used in nanophotothermal platforms to produce nanocapsules or coat nanoparticles, thereby enhancing their circulation time and uptake.	(35, 44, 45)
Hyaluronic Acid (HA)	Native, long-strand HA is known for its ability to reduce immune rejection, i.e., to act as an immune-calming agent that suppresses harmful inflammation and promotes healing. A key advantage of HA is its ability to actively target CD44 receptors, which are overexpressed on many cancer cells. Nanoparticles coated with HA achieve precise accumulation at tumor sites for both chemotherapy and photothermal therapy.	(46–48)
Alginate	Biopolymer that is valued for its biocompatibility and ability to form robust scaffolds for drug delivery.	(20)
Cellulose	Often used in the form of carboxymethyl cellulose (CMC) to improve the stability, compatibility, and dispersibility of nanocarriers.	(22)
Polyphenolic and Bio-inspired Organic Polymers—a new category for materials derived from natural pigments or complex organic structures		
Polydopamine (PDA)	Referred to as a synthetic melanin, is highly valued in nanophotothermal systems for its strong broadband NIR absorption and exceptional biocompatibility	(34, 43)
Lignin	A complex natural organic polymer used in nanoconposites to provide antioxidant properties and act as a stabilizing coating for magnetic and metallic nanoparticles.	(23)
Proteins and Protein-based Scaffolds—offer excellent biocompatibility and high densities of functional groups for drug loading		
Albumin	Used as stabilizing scaffolds or matrices that improve the solubility and stability of photothermal agents like indocyanine green (ICG)	(26)
Gelatin	Creates thermosensitive hydrogels or nanosphere matrices with uniform drug distribution	(25)
Silk fibroin	Used in tissue regeneration and PTT platforms gives mechanical strength and tunable biodegradability of DD systems.	(42)
Biomembrane-based (biomimetic) systems—These systems utilize or mimic natural cell structures to bypass the immune system and achieve targeted delivery.		
Liposomes	Primarily composed of natural phospholipids. Their lipid bilayer structure mimics that of natural biological membranes. They can be loaded with both hydrophilic and hydrophobic drugs. Engineered to be thermosensitive, liposomes disintegrate and release their loaded drug specifically under NIR light.	(3, 8, 49)
Cell Membranes	Natural multifunctional materials extracted from red blood cells, cancer cells, or immune cells to “cloak” nanoparticles, providing them with innate homing abilities and longer circulation times	(40)
Exosomes	Naturally occurring extracellular vesicles (30–100 nm) that possess a lipid bilayer and a unique surface protein profile, enabling them to penetrate biological barriers and penetrate deeply into tumor tissue	(40)

Given the breadth of this rapidly expanding field, an exhaustive material-by-material analysis is beyond the scope of the present review. Instead, the following sections focus on chitosan and hyaluronic acid as two representative polysaccharide-based platforms that illustrate complementary structure–function strategies in smart nanophotothermal drug delivery.

### 3.1 Chitosan as a nanophotothermal drug carrier

Alkaline polycationic polysaccharide, chitosan (CS, is the naturally occurring biopolymer derived from the deacetylation of chitin. Randomly distributed *β*(1→4)-linked D-glucosamine and N-acetyl-D-glucosamine units, along with amino groups (NH_2_), give chitosan a unique combination of biocompatibility, biodegradability, and non-toxicity (44). Due to the presence of many functional groups (NH_2_ and OH), chitosan is amenable to different functionalization strategies (44), which in turn facilitate the development of systems such as nanoparticles, hydrogels, and three-dimensional scaffolds for targeted drug delivery and tissue engineering. It was shown that, in addition to providing structural support, chitosan enhances cellular uptake through electrostatic interactions with negatively charged cell membranes and promotes prolonged drug retention at tumor sites (21). Consequently, it has become a key component in numerous multifunctional nanoplatforms designed for precision cancer therapy (21, 50).

Chitosan-based photothermal platforms use different preparation strategies, including self-assembly and electrostatic interactions, surface stabilization/coating, *in situ* hydrogel formation, and nanoscale fabrication. Electrostatic interactions between cationic amino groups in CS and anionic species, such as HA or specific drugs, lead to the formation of nanostructures. Jo et al. (51) prepared a nanocomplex based on chitosan oligosaccharide lactate conjugated to a zwitterionic dye and hyaluronic acid (HA), resulting from electrostatic interactions between the positively charged -NH_2_ groups of chitosan and the negatively charged -COOH groups of HA. CS is also used for encapsulation or coating inorganic photothermal agents, such as gold nanoparticles (52), graphene oxide, graphene quantum dots (35), MXenes (50), polydopamine (53), Prussian blue (54), magnetic nanoparticles (55), metal–organic frameworks (56), mesoporous silica (57), which prevent their clustering and improve biocompatibility and stability. Injectable platforms, prepared by mixing CS and sodium *β*-glycerophosphate as a crosslinker with carbon nanoparticle suspensions containing lymph node tracers, when injected into the body, and, upon reaching 37 °C, undergo a liquid-to-gel transition, forming hydrogels via physical crosslinking (58). On the other hand, by means of dynamic covalent bonding (e.g., Schiff base bonds), Guo et al. (59) prepared a multifunctional nanocomposite system with CS as the matrix, encapsulating nanoparticles loaded with the pH-responsive camptothecin and the photothermal agent polydopamine, which was shown to be efficient in the treatment of uveal melanoma. Techniques such as electrospinning and 3D bioprinting are used to fabricate nanoscale fiber structures with high surface-area-to-volume ratios that mimic the natural extracellular matrix and are used, for example, in bone cancer treatments and bone recovery (44, 60).

Incorporation of therapeutics into chitosan nanoformulations can be achieved via physical entrapment and encapsulation (61), self-assembly and electrostatic interactions (51), and adsorption (57). But, the transition from passive to active targeting, which enhances receptor-mediated uptake and tumor selectivity, is achieved through chitosan surface functionalization with targeting ligands such as hyaluronic acid (61), biotin (62), aptamers (35), or RGD peptides (56).

“Smart” drug delivery mechanisms of chitosan platforms for controlled drug discharge include pH- and thermo-responsiveness, redox sensitivity (21, 42, 56), and endosomal escape (61). Namely, the protonation of amino groups in the tumor acidic microenvironment increases the swelling and the solubility of the chitosan matrix and facilitates local drug release (52). Slow drug delivery from chitosan thermo-nanogels is achieved via the sol-gel transition induced by physiological heat or localized hyperthermia from NIR irradiation (43). Therapeuticals attached via disulfide bonds to the chitosan nanosystems are safely transported through the bloodstream to cancer cells, where high levels of glutathione (GSH) attack and cleave the disulfide bonds, releasing the drug (59). The cationic nature of CS, which carries the drug, facilitates endosomal escape via the proton-sponge effect, thereby enhancing cytosolic drug delivery (61).

Typically, chitosan itself doesn't exhibit light absorption at NIR wavelengths, but it serves as an ideal matrix or support for incorporating NIR-responsive agents. Namely, the optimized spacing between nanopagents provided by the CS matrix prevents their clumping and yields a larger surface area for interactions with exogenous stimuli, e.g., NIR light. CS-based nanocomposites under NIR laser irradiation can elevate local temperatures within minutes to certain therapeutic ranges (59). Thus, a thermogel prepared from mesoporous doxorubicin-loaded polydopamine nanoparticles and hexanoyl glycol chitosan, under 808 nm NIR laser irradiation, exhibited a synergistic chemotherapeutic effect in the treatment of intraocular cancer (43). Innovative PPT designs, such as photothermoelectric cobalt-chitosan nanocomposites, act as self-regulating thermostats. The electric signal generated when they are heated offers real-time temperature monitoring and precise temperature regulation (63).

As sophisticated theranostic hubs, chitosan-based platforms employ diverse therapeutic approaches to target tumors while simultaneously promoting tissue repair, which are divided into two main groups: integrated therapeutic methods and tissue engineering approaches (44). Integrated therapeutic modalities include photodynamic and chemodynamic therapy, magnetic hyperthermia, radiotherapy and radiosenzation, starvation therapy, ferroptosis induction, and immunotherapy (42, 44, 54, 64). The design of “smart” systems enables sequential therapeutic activation that responds to changes in environmental pH (52, 57), finds and adheres to the diseased tissue, breaks down using redox reactions to fight cancer from inside (21, 59), and, with the implementation of exogenous stimuli, triggers a final, powerful dose of medicine, ensuring that treatment is fine-tuned to the evolving state of the disease (43, 57). In photodynamic therapy (PDT), chitosan nanoparticles and hydrogels encapsulating photosensitizers (such as ICG or zwitterionic NIR dyes) are used with NIR light (43, 51, 56). When the dye absorbs energy, it reacts with oxygen in the body, generating reactive oxygen species that destroy cancer cells’ membranes, causing their extinction (40). Chemodynamic therapy (CDT) involves an acidic tumor microenvironment, which causes chitosan to swell and release metal ions (e.g., iron, zirconium, or manganese), which act as nanozymes and kick off the Felton reaction of converting H_2_O_2_ into hydroxyl radicals (•OH), destroying cancer cell walls and cancer DNA, killing the cancer without harming normal tissues (53). Magnetic hyperthermia can be applied to claster-shell systems in which chitosan, as a nanomatrix, embeds magnetic or supermagnetic iron oxide nanoparticles (55). With the alternating magnetic field, these platforms can be guided to the tumor site, where they generate localized heat (55), which is particularly effective for treating deep-seated tumors where NIR light penetration is limited (44). Chitosan-based scaffold and hydrogel systems containing radiosensitizers such as gold nanoparticles or cisplatin improve x-ray dose deposition directly within the tumor but also allow for lower radiation doses to achieve effective disease control (57). Multifunctional chitosan carriers can co-encapsulate enzymes such as glucose oxidase (GOx). The effect of these systems is achieved by oxidizing intratumoral glucose, thereby depleting the tumor's energy supply and generating H_2_O_2_ as a byproduct, supporting CDT and enhancing the overall therapeutic outcome (54). Nanozyme-hybrid chitosan platforms facilitate ferroptosis, a regulated cell death driven by the iron-dependent lipid peroxides, a toxic fat build-up in the cell membranes. These systems work by depleting antioxidant defenses (such as glutathione) and inducing extensive oxidative damage, which is especially effective against cancer cells resistant to multiple drugs (64).

In tissue engineering, due to its structural resemblance to extracellular matrix glycosaminoglycans, chitosan serves as an exceptional scaffold for bone and cartilage regeneration (21, 44). In addition to encapsulating PTAs, which cause hyperthermia and increase cellular permeability, the same chitosan matrix can be used to deliver chemotherapeutic drugs or radiosensitizers. Melo et al. (45) achieved synergistic chemo-photothermal therapy for breast cancer using a chitosan hydrogel containing dopamine-reduced graphene oxide, a photothermal nanoagent, and resveratrol, a chemotherapeutic drug. As shown by Shi et al. (57), a multi-responsive platform consisting of chitosan/acellular fish skin hydrogel matrix embedding core–shell gold/mesoporous silica nanoparticles, functionalized with a pH/ROS-responsive linker and hyaluronic acid, and loaded with 5-fluorouracil (5-FU), was a successful therapeutic for colorectal cancer.

Despite the advantages of CS related to biocompatibility and safety (44) mucoadhesion (64), stabilization and inherent bioactivity (21), chitosan faces many drawbacks, which might be related to nonspecific protein adsorption and its rapid clearance from the bloodstream (45, 51).

### 3.2 Hyaluronic acid as nanophotothermal drug carrier

Hyaluronic acid (HA) is a natural mucopolysaccharide consisting of alternating disaccharide units of D-glucuronic acid and N-acetyl-D-glucosamine linked by β-1, 3 and β-1, 4 glycosidic bonds. As a major constituent of the extracellular matrix, HA acts as a super sponge in the living body (46, 48, 65). Excellent biocompatibility, biodegradability, non-toxicity, and non-immunogenicity grant high value to nanomedicine applications of HA, drawing particular attention to its roles in tissue hydration, cell adhesion, proliferation, migration, wound healing, and extracellular signaling (48, 66, 67).

The use of HA in DDSs is primarily driven by its ability to interact with several cell-surface receptors, commonly cluster of differentiation 44 (CD44) (68, 69), which, upon binding, enter malignant cells, resulting in their increased intracellular accumulation (70, 71), reduced systemic toxicity, and higher cancer therapeutic index (72, 73).

The chemical structure of HA enables various chemical functionalizations, mainly through carboxyl groups on the glucuronic acid unit and hydroxyl groups on the N-acetyl-D-glucosamine unit (48, 73), and further covalent conjugation of HA to drugs (e.g., methotrexate) (24), imaging agents (51), targeting ligands, photoresponsive nanomaterials (73), or other polymers (e.g., PLGA or chitosan) (61).

The HA-based smart systems achieve precise control of drug delivery not only through active targeting to the tumor site but also by responding to endogenous or exogenous factors. The synergistic combination of these stimuli enables selective drug release only after nanoparticle accumulation within tumors, thereby minimizing premature drug leakage before the nanocarrier reaches the target site (74, 75). Internal stimuli include acidic pH and other internal signals, such as increased concentrations of reactive oxygen species (ROS) or disease-specific molecules (e.g., glutathione), as well as enzymatic degradation within the tumor microenvironment (24, 74). Exogenous triggering commonly includes near-infrared (NIR) laser irradiation (75).

“Smart” nanoplatforms can leverage the acidic nature of the tumor microenvironment as an internal stimulus to achieve precise drug delivery. When getting in contact with cancer cells, which, due to high glycolytic metabolism and lactic acid accumulation, have an acidic pH of ∼6.5, nanocarriers encounter even more acidic environments within endosomes and lysosomes (pH ∼4.0–5.0) (65, 76). Two primary mechanisms underlie the pH stimulus that triggers drug release from HA platforms. The first concerns acid-sensitive chemical linkers such as hydrazone and acylhydrazone bonds (47), ester bonds (76), and dynamic imine (Schiff base) bonds (42), while the second mechanism involves physical destabilization and swelling of the nanostructures (46, 70). Hydrazone and acylhydrazone bonds are widely used to link chemotherapeutic drugs such as DOX (47) and ester bonds for dasatinib (DAS) (76). In many hybrid systems, such as CS–HA nanoparticles, pH changes trigger a physical transformation, i.e., destabilization and swelling, of the nanocarrier, allowing the encapsulated drug to escape more easily (70). HA-coated mesoporous silica nanoparticles exhibit so-called gatekeeping mechanisms, where the HA shell acts as a pH-responsive gatekeeper. At neutral pH, HA blocks the silica pores, preventing drug leakage. In the acidic tumor environment, the HA “gate” is compromised and opens, initiating drug release (57). Systems of gold nanorings modified with HA and loaded with the drug DOX showed only ∼20% DOX release at pH 7.4, whereas at pH 5.0, this percentage increased to ∼50%. Further treatment with the NIR laser induces localized heating, thereby maximizing therapeutic efficacy (77).

On the other hand, ROS-responsive systems utilize linkers such as thioketal moieties or oxide-sulfide bonds that are covalently bonded to HA and undergo rapid cleavage and drug release upon encountering high ROS concentrations within malignant tissue (24, 46), in which high levels of reactive oxygen species enable the system to recognize the tumor's “fingerprint”. Beyond synthetic linkers, HA nanoparticles can incorporate a bilirubin core. It is also noted that the HA polymer itself is susceptible to nonspecific degradation via oxidative damage induced by ROS, which usually results in breakdown of the nanostructure and the release of its therapeutic cargo (48).

Glutathione (GSH)-responsive systems are a prominent class of redox-sensitive nanoplatforms that exploit the GSH concentration difference between the extracellular (2–20 µM) and intracellular (2-10 mM) environments (78). Various polymer-drug and polymer-polymer conjugates (46, 75) utilize GSH-responsiveness for precise delivery. E.g., HA-conjugated gemcitabine (GCB) or metformin (MET) via disulfide bonds in a triple-responsive system (pH/GSH/thermal) selectively accumulate in tumors via CD44 targeting and release their cargo in response to intracellular GSH (75).

Some HA-based nanoformulations use enzymes secreted by tumor cells, e.g., matrix metalloproteinases (MMPs), such as MMP-2 and MMP-9, to achieve targeted drug delivery. E.g., by inserting an MMP9-cleavable peptide linker between the Polyethylene glycol (PEG) and the HA backbone, the PEG corona is detached only at the tumor site, exposing the HA for CD44-mediated uptake (46).

For greater precision in drug delivery, multi-responsive systems combine internal and external triggers, e.g., dual pH/NIR (77) and pH/ROS (78) or triple pH/GSH/thermal (59). Ding et al. (79) showed the synergistic chemo-phototherapy of an HA-modified nano-delivery system incorporating ivermectin and a phototherapeutic agent (IR780-derived compound, H780). Following intravenous administration every two days (during a 15-day study) and 660 nm laser irradiation (1 W/cm^2^, 3 min), the observed tumor inhibition rate in the CT26 colorectal cancer model in BALB/c mice (*n* = 5/group) was 92.4%.

Combining HA with gold nanostructures (74), polydopamine (75), mesoporous silica (80), magnetic nanoparticles (81, 82), and other photothermal agents (83) enables the integration of active targeting with efficient photothermal conversion and multimodal therapeutic function. The integration of HA into multifunctional nanophotothermal platforms has substantially expanded the scope of multimodal cancer therapy. Most recent systems combine PTT with chemotherapy (CT), although combinations involving PDT, gene therapy, CDT, immunotherapy, and diagnostic imaging are becoming increasingly important in achieving superior therapeutic outcomes (47, 72, 73).

Thus, smart drug delivery of HA-modified gold nanostructures, where HA provides the system's colloidal stability and active targeting, while photothermal therapy under near-infrared (NIR) light is enabled by the metal's optical properties (47). Hu et al. (77) investigated an AU nanoring system modified with HA and loaded with the anticancer drug DOX for the treatment of pancreatic tumors in mice. It was observed that the metal's photothermal effect heats cancer sites and, in combination with pH-driven drug release in the microenvironment, leads to greater suppression of cancer cells than a single therapy. Similarly, Zeng et al. (80) developed a nanoplatform composed of polydopamine (PDA)-encapsulated DOX and HA-modified mesoporous silica nanoparticle (MSN), which was tested in orthotopic 4T1 breast tumor-bearing mice (*n* = 3/group). After intravenous administration of the system and NIR irradiation (808 nm, 2 W/cm^2^, 10 min), the scientists observed a 72% decrease in cancer volume, confirming the potential of using the synergistic effect of photothermal and chemotherapy treatments. To emphasize the photothermal performance of nanomedicines in cancer therapies and to provide multiple functional, a hybrid HA-gold/Polydopamine/Dox has been developed (74). Addressing the difficulties related to drug delivery to the exact location, Yun et al. (24) prepared an HA–Methotrexate (MTX) conjugate, i.e., a smart nanoplatform that connects the chemotherapy drug Methotrexate (MTX) to HA via ROS stimulus-responsive thioketal linker. The hydrophobic IR780 photothermal molecule is “hiding” within the hydrophilic nanoparticles to remain stable. In addition to targeted drug delivery, these formulations achieve an inhibition rate of around 71% in 4T1 tumor cells. These examples indicate that HA-based nanophotothermal systems are not only passive drug carriers, but they also improve tumor recognition, selective cellular uptake, and therapeutic precision (46, 67).

Real-time monitoring of drug delivery and the tumor's therapeutic response is boosted by the development of theranostic platforms that integrate HA into multifunctional nanoparticles and combine targeted therapy with imaging modalities such as fluorescence, photoacoustic imaging, and magnetic resonance imaging (MRI) (73, 77). Such approaches expand the scope of multimodal cancer therapy toward personalized precision oncology. Loading nanoparticles with NIR dyes such as DiR or FITC, or leveraging the intrinsic fluorescence of drugs such as DOX, enables high-sensitivity visualization of drug cellular uptake and intracellular drug release (40, 77, 78), particularly knowing that HA-mediated endocytosis concentrates these agents within the tumor cells (77). HA-based coated platforms containing MRI contrast agents, e.g., superparamagnetic iron oxide nanoparticles (SPIONs) and chelating ions such as manganese (Mn^2^⁺) or gadolinium, provide detailed anatomical information. Usually, these systems are typically used in bimodal imaging, combining, for example, MRI and fluorescence, to compensate for the limitations of each modality (72, 73).

All of the above indicate that HA nanosystems offer a sophisticated “all-in-one” platform for synergistic targeted cancer therapy that overcomes the limitations of conventional treatments (67, 72). In addition to the many advantages of HA-based nanophotothermal systems, including inherent biocompatibility, biodegradability, and safety profile (47, 70), several challenges continue to limit their clinical translation, including biological and targeting limitations, manufacturing and industrial scale-up, *in vivo* stability, translational gaps, safety, and regulatory limitations.

Consequently, the development of standardized HA nanosystems with controllable molecular architectures, programmed multi-stimuli responsiveness, and integrated theranostic capabilities should be the focus of future scientific research.

## 4 Comparative structure–function perspectives of chitosan and hyaluronic acid

In the engineering of bio-based nanophotothermal drug delivery systems, the two most frequently used complementary bio-polymers are chitosan and hyaluronic acid. The selection of polymers and/or their combinations is not based on the intrinsic superiority of one material over another, but should be guided by the intended function of the nanophotothermal platform. Thus, the chitosan's primary function is as a chemically adaptable engineering matrix, whereas hyaluronic acid serves as the biologically active targeting matrix (51).

Distinctions between these two biomaterials are given in Table 2 (A). Basically, differences in their structure, specifically their ionic nature and different functional groups, dictate divergent and complementary roles in targeted drug delivery and photothermal therapy. Representative quantitative performance data for selected chitosan- and hyaluronic acid-based NPT DDSs are also presented in Table 2 (B).

**Table 2:** Part A. Comparative structure–function characteristics of chitosan and hyaluronic acid in smart NPT DDSs. Part B. Representative quantitative performance data for selected chitosan- and hyaluronic acid-based NPT DDSs.

Part A			
Design parameter	Chitosan	Hyaluronic acid	Ref.
Ionic character	Cationic; protonatable amino groups confer pH-dependent charge	Anionic; negatively charged carboxyl groups	(24, 32, 44–46, 48, 51, 58–60, 67, 68, 71, 74, 75, 80, 84)
Major functional groups	Amino (–NH₂) and hydroxyl (–OH) groups	Carboxyl (–COOH) and hydroxyl (–OH) groups	
Principal design role	Chemically versatile engineering matrix	Biologically active targeting matrix/ligand	
Key smart property	pH responsiveness associated with amino-group protonation; readily engineered for thermo-, redox-, and multi-stimuli responsiveness	Intrinsic receptor recognition, particularly CD44-mediated targeting; readily modified for pH-, ROS-, redox-, and enzyme-responsive release	
Nanoplatform formation	Nanoparticles, coatings, polyelectrolyte complexes, injectable thermogels, hydrogels, scaffolds, nanofibres	Nanoparticles, surface coatings, polymer–drug conjugates, amphiphilic micelles, nanogels, hydrogels, targeted shells	
Drug-loading strategy	Physical entrapment, encapsulation, electrostatic complexation, adsorption and chemical conjugation	Covalent drug conjugation, amphiphilic self-assembly, encapsulation, surface coating and incorporation of cleavable linkers	
Photothermal role	Primarily a carrier/support for NIR-responsive agents; stabilizes and spatially confines photothermal components	Primarily a targeting/coating component surrounding photothermal cores; directs NIR-responsive systems toward receptor-positive tumor cells	
Local delivery potential	Particularly strong; thermosensitive hydrogels, mucoadhesive depots and 3D scaffolds enable sustained localized treatment	Hydrogels and nanogels support localized delivery, although HA is particularly prominent in targeted systemic nanocarriers	
Tumor targeting	Usually requires an added ligand, e.g., HA, biotin, aptamer or RGD peptide	Intrinsic affinity for HA-binding receptors, predominantly CD44; RHAMM and other receptors may also contribute	
Cellular internalization	Electrostatic interaction with negatively charged membranes; cationic character may facilitate endosomal escape	Receptor-mediated endocytosis following HA–CD44 interaction	
TME-responsive behaviour	Native pH sensitivity; additional redox, ROS, enzyme and thermal responsiveness introduced through chemical engineering	Responsiveness generally engineered through acid-labile, thioketal, disulfide or enzyme-cleavable linkages; HA itself is susceptible to hyaluronidase-mediated degradation	
Major multimodal strengths	Local chemo–PTT, CDT–PTT, magnetic hyperthermia, radio–PTT, starvation therapy, ferroptosis, immunotherapy and regenerative scaffolds	Targeted chemo–PTT, PDT–PTT, gene delivery, immunotherapy, theranostics and multi-stimuli-responsive precision therapy	
Particular advantage	Material programmability, mucoadhesion, hydrogel/scaffold formation and versatile cargo integration	Biological selectivity, receptor-mediated targeting and intracellular accumulation	
Main limitations	Molecular-weight/deacetylation variability, physiological solubility, nonspecific interactions of cationic surfaces, and mechanical limitations	Molecular-weight-dependent biological effects, CD44/receptor heterogeneity, hyaluronidase degradation, and possible loss of receptor affinity after extensive modification	
Complementary role in hybrid CS–HA systems	Provides structural matrix, drug entrapment, pH responsiveness and intracellular delivery functions	Reduces excessive cationic surface character and provides receptor-mediated tumor recognition and targeting	

Part B					
Nanoplatform/ therapeutic cargo	Experimental model and relevant conditions	Tumor accumulation/ uptake	Drug-release kinetics	Cytotoxicity threshold/ quantitative outcome	Ref.
CS-based DOX-Fe₃O₄@CS@GQD-Apt (DOX-Fe₃O₄@CGA); DOX	H22 hepatoma cells; H22 tumor-bearing mice; NIR: 808 nm, 2 W/cm^2^, 5 min	Method: *in vivo* fluorescence imaging using ICG-labelled nanoparticles, with fluorescence-intensity analysis of the tumor region at 2, 6, 24, and 48 h. *In vivo* fluorescence increased at the tumor site, peaking at 24 h after injection and declining at 48 h; accumulation percentage not reported.	*In vitro* dialysis release study. DOX-Fe₃O₄@CGA/PBS/37 °C/PBS/ dialysis bag/pH 5.5 or 7.4/stirring/with or without NIR (808 nm, 2 W/cm^2^, 5 min). At pH 5.5 + NIR, DOX release reached ∼70% after 30 h, versus ∼20% at pH 7.4 without NIR.	*In vitro*. MTT assay. Cells seeded in 96-well plates, exposed to Fe₃O₄@CGA at different concentrations, exposed to NIR, 24 h incubation. At a DOX concentration of 20 µg/mL, DOX-Fe₃O₄@CGA + NIR reduced cell viability to 37%. At 800 µg/mL unloaded carrier maintained >70% cell survival.	(35)
CS-based NIOs/AuNPs-CS/ARI; ARI	MCF-7 breast cancer cells; ARI tested at 12–96 µM, 24 and 48 h; combined treatment used 48 µM ARI	Method: fluorescence-microscopy-based cellular uptake assessment in MCF-7 cells. Cellular uptake of NIOs/AuNPs-CS was significantly greater than blank NIOs; no numerical accumulation percentage reported.	*In vitro* dialysis release study. NIOs/AuNPs-CS/ARI/37 °C/PBS/dialysis bag/pH 5.8 or 7.4/stirring/, and agitated at 90 rpm. ARI quantified by UV–Vis at 250 nm. pH-responsive release; best fitted by Power-law/Weibull models. For NIOs/AuNPs-CS/ARI, Power-law R^2^ = 0.9866 (pH 5.8) and 0.922 (pH 7.4); release was predominantly Fickian diffusion-controlled.	*In vitro*. MTT assay, *n* = 4 mice/group. Cells treated with: free ARI, NIOs, ARI/NIOs, NIOs/AuNPs-CS, or NIOs/AuNPs-CS/ARI at 12, 24, 48, and 96 µM, 24 or 48 h. (*n* = 4/group) ARI-loaded nanoparticles showed IC₅₀ ≈ 48 µM after 48 h. At 48 µM, NIOs/AuNPs-CS/ARI + PTT produced approximately 60% cell death at 24 h and 80% at 48 h.	(52)
CS-based Ch@Au-MXNSs@Cur; curcumin	MCF-7 cells; curcumin-loaded Au-MXene coated with CS; NIR: 808 nm, 1 W/cm^2^, 5 min	Method: *in vivo* antitumor evaluation Antitumor activity demonstrated, but quantitative tumor accumulation was not reported.	*In vitro* dialysis release study. Ch@Au-MXNSs@Cur PBS/37 °C/dialysis bag/pH 5.5 or 7.4/shaking/NIR. Curcumin quantified by spectrofluorometry for 72 h. Curcumin release after 72 h: 50.5% at pH 5.5 versus 10.1% at pH 7.4; after 24 h: 38.8% vs. 7.2%, respectively.	*In vitro*. MTT assay. Cells cultured in DMEM supplemented with FBS, penicillin/ streptomycin, 37 °C and CO₂. NIR applied, followed by 48 h incubation. IC₅₀ values: 41.97 µg/mL for chemo-PTT, 58 µg/mL for PTT, 73.28 µg/mL for chemotherapy, and 83.75 µg/mL for nanocomposite alone.	(50)
CS-based HGC-MPDA@DOX thermogel; DOX	OCM-1 intraocular cancer cells; local thermogel; MPDA 200 µg/mL, DOX 148.5 µg/mL; NIR 808 nm, 1.5 W/cm^2^	Local administration/*in situ*-forming thermogel; systemic tumor accumulation efficiency not applicable/reported.	*In vitro* release study from the thermogel. DOX release monitored with and without 808-nm NIR, using a standard-curve quantification method. At 120 min, cumulative release was 10.0 ± 0.4% with NIR vs. 8.0 ± 0.3% without NIR, corresponding to an approximately 25% irradiation-induced increase.	*In vitro*. CCK-8 assay in a Transwell co-culture system. Cells seeded at 2 × 10⁴ cells/well in 24-well plates, cultured for 24 h. Cells were co-cultured with thermogel for 48 h. PTT groups received NIR irradiation. Combined HGC-MPDA@DOX + NIR reduced OCM-1 viability to 17.5 ± 1.3%, compared with 63.9 ± 7.6% for PTT alone and 32.6 ± 3.9% for chemotherapy alone.	(43)
HA-based HA/DOX-AuNRs; DOX-loaded gold nanorings	Panc-1 pancreatic cancer cells and BALB/c nude mouse Panc-1 xenografts; IV administration; NIR-II 1064 nm, 2 W/cm^2^	Method: serial *in vivo* fluorescence imaging and photoacoustic imaging, with quantitative gold determination in excised tumor tissue by ICP-MS at 24 h. Tumor accumulation efficiency was 11.3%, approximately 3-fold higher than AuNRs; fluorescence/PA signals persisted to 36 h.	*In vitro* dual-responsive release study. DOX release compared at pH 5.0 and 7.4, with and without NIR-II irradiation. Approximately 50% DOX release at pH 5.0 within 8 h, compared with ∼20% at pH 7.4; NIR-II-induced heating further accelerated release.	*In vitro*. CCK-8 assay. Cells were incubated with HA/DOX-AuNRs, AuNRs, or DOX for 24 h, irradiated with NIR-II, and incubated for 24 h, *n* = 6 mice/group. Cell viability decreased markedly once the equivalent Au concentration exceeded 20 µg/mL.	(77)
HA-based DOX/MPH: MSN@PDA@HA/ DOX	4T1 and MDA-MB-231 breast cancer cells; 4T1 orthotopic mouse model; NIR 808 nm, 2 W/cm^2^, 10 min	Method: quantitative flow cytometry using intracellular DOX mean fluorescence intensity (MFI), together with an HA competitive CD44 receptor-blocking assay; *in vivo* tumor localization was additionally assessed indirectly by tumor-region thermal imaging. Dox/MPH produced significantly greater MFI than non-HA Dox/MP (*p* < 0.0001), whereas excess free HA reduced uptake to approximately the non-targeted baseline; no tumor accumulation percentage was reported.	*In vitro* release study. Nanoparticle dispersion/PBS/37 °C/pH 5.0 or 7.4/stirred. Samples were analyzed at 1, 2, 4, 8, 12, 24, and 48 h by UV–Vis. For the NIR group, nanoparticles were first irradiated at 808 nm, 2 W/cm^2^ for 10 min. Release followed the Korsmeyer-Peppas model (R^2^ > 0.96); NIR significantly accelerated release (f₂ < 50).	*In vitro*. CCK-8 assay, *n* = 3 mice/group) Cells treated with MPH or DOX/MPH. After 2 h incubation, NIR applied, incubation for 24 or 48 h at 37 °C. Under NIR, when MPH concentration exceeded 60 µg/mL, viability of 4T1/MDA-MB-231 cells fell below 20%. Normal L929 viability remained >80% at equivalent DOX 10 µg/mL without NIR.	(80)
HA/CS-hybrid HA-COL-ZW phototheranostic nanocomplex; ZW800-1	NCI-H460 tumor-bearing mice; IV 10 nmol/100 µL based on ZW800-1; NIR 808 nm, 1 W/cm^2^, 5 min	Method: serial *in vivo* NIR fluorescence imaging for 24 h, followed by *ex vivo* fluorescence analysis of resected tumors and organs and extended biodistribution/clearance monitoring to 96 h. HA-COL-ZW showed strong tumor accumulation maintained to 24 h, with renal clearance and essentially complete clearance by 96 h; no accumulation percentage was reported.	Not applicable. ZW800-1 is covalently incorporated into the HA/COL phototheranostic nanocomplex; this system was not evaluated as a conventional chemotherapeutic drug-release formulation.	*In vivo*. PTT efficacy. Following intravenous administration and 24 h accumulation, NIR irradiation (808 nm, 1 W/cm^2^, 5 min) (*n* = 3/group) increased tumor temperature to 55.5 °C versus 34.9 °C in PBS-treated controls.	(51)
HA/CS-hybrid HA-CS-PLGA-DOX/ICG NPs; DOX + ICG	MDA-MB-231 TNBC cells; TNBC xenograft model; NIR 808 nm, 1.5 W/cm^2^, 5 min	Method: CLSM visualization of intracellular DOX/ICG combined with flow cytometry for quantitative cellular uptake. HA functionalization produced a 2.3-fold increase in cellular fluorescence/uptake versus unmodified PLGA after 4 h; not reported as % tumor accumulation.	*In vitro* dialysis release study. HA-CS-PLGA-DOX/ICG/37 °C/PBS/ dialysis bag/pH 5.5 or 7.4. DOX/ICG quantified spectrophotometrically, at intervals for 72 h, At 72 h, DOX release was 94.6 ± 2.3% at pH 5.5 vs. 69.4 ± 2.1% at pH 7.4; ICG release was 89.8 ± 2.1% vs. 61.2 ± 1.9%, respectively.	*In vitro*. MTT assay, *n* = 6 mice/group. Cells seeded at 1 × 10⁴ cells/well, exposed for 24 or 48 h to free DOX (5 µg/mL), free ICG (4 µg/mL) + NIR, DOX/ICG nanoparticles, or HA-CS-PLGA-DOX/ICG NIR-treated groups were irradiated with 808 nm light at 1.0 W/cm^2^ for 5 min. The combined HA-CS-PLGA-DOX/ICG + NIR treatment reduced viability to 25.1 ± 1.3% at 24 h and 13.5 ± 1.0% at 48 h.	(61)

Quantitative values are formulation- and assay-specific and should not be interpreted as direct evidence of superiority of one biopolymer or nanoplatform over another. Tumor accumulation/uptake was evaluated using different methodologies, including ICP-MS, *in vivo* fluorescence imaging, photoacoustic imaging, CLSM, flow cytometry, and biodistribution analysis. Tumor accumulation efficiency (%) was not explicitly reported in most of the papers. Drug-release and cytotoxicity results should be interpreted in relation to the specific experimental conditions, including pH, temperature, incubation time, drug/nanoparticle concentration, biological model, and irradiation parameters.

Apt, aptamer; ARI, aripiprazole; AuNPs, Gold Nanoparticles; Ch, CS, Chitosan; COL, chitosan oligosaccharide lactate; Cur, curcumin; DOX, doxorubicin; GQD, graphene quantum dots; HA, hyaluronic acid; HGC, hexanoyl glycol chitosan; ICG, Indocyanine Green; MPDA, mesoporous polydopamine nanoparticles; MSN, mesoporous Silica Nanoparticles; MXNSs, MXene Nanosheets, 2D transition metal carbides/carbonitrides; NIOs, niosomes; PDA, polydopamine; PLGA, poly(lactic-co-glycolic acid); ZW, zwitterionic; ZW800-1, zwitterionic near-infrared dye.

Chitosan is rich in amino and hydroxyl groups, which facilitate its chemical conjugation to thermo-, redox-, ROS-, or enzyme-responsive ligands, responsible for its environment-responsive physicochemical behavior. Additionally, the protonation of the amino groups under acidic conditions contributes to pH-responsive swelling of chitosan nanomatrices and drug release. Chemical modification of chitosan matrix converts the natural polymer into “smart” therapeutic material such as thermosensitive nanoplatforms, injectable hydrogels, redox-responsive nanocomposites, and photothermoelectric feedback systems (59, 62, 63). Similarly, the carboxyl and hydroxyl functionalities in HA provide sites for conjugation with various agents, including drugs, stimuli-responsive linkers, imaging probes, and other species. In contrast to chitosan, the defining “smart” property of HA is biological recognition, i.e, interaction with several cell-surface receptors, particularly CD44 and RHAMM, which promote receptor-mediated endocytosis and increase intracellular nanocarrier accumulation (68, 69).

Differences in the chemical reactivity and ionic character of chitosan and HA influence the drug-loading and delivery strategies of their respective nanosystems. Chitosan is particularly effective in physical entrapment of small-molecule drugs, photosensitizers, nucleic acids, enzymes, and inorganic photothermal components. Interactions with negatively charged nucleic acids and contribution to intracellular delivery, including endosomal escape, are supported by the cationic character of chitosan (51, 59, 62). In contrast, HA can participate as an outer shell for active drug targeting, as a drug-polymer conjugate, or an amphiphilic self-assembly system. Connecting HA to various species, such as ROS-, glutathione-, or pH-responsive agents, via acid-labile linkages enables these systems to release therapeutics in response to the given stimuli (24, 74, 75). This architecture is particularly advantageous when active tumor targeting and controlled intracellular release must be integrated into a single platform.

Chitosan and HA also differ in their suitability for local systemic drug delivery. Regarding its mucoadhesive properties and capacity to form thermosensitive hydrogels, injectable matrices, and three-dimensional scaffolds, chitosan is suitable for use as a localized therapeutic depot. Generally, chitosan-based systems retain therapeutic and photothermal agents at the cancer or post-resection site, reduce systemic exposure, and provide sustained or externally activated treatments. Chitosan-based nanohydrogels and nanoparticles have therefore been explored for breast, colorectal, bladder, intraocular, and bone cancer applications, including combined tumor elimination and tissue regeneration (45, 58, 59, 70). Despite its similar ability to form hydrogels and nanogels, the main difference of HA, as far as drug delivery is concerned, is its role as a systemically administered targeted nanocarrier, in which receptor recognition supports tumor accumulation and intracellular uptake (24, 75, 84). Yet, the distinction between local and systemic application is not absolute, as evidenced by the possibility of HA for mucoadhesive and localized delivery in specific cases, particularly in colorectal cancer (66).

Considering the tumor-targeting perspective, HA has a clear intrinsic biological advantage over chitosan. Namely, due to the lack of specific tumor receptors, pure chitosan needs functionalization with targeting ligands such as HA (61), biotin (62), RGD peptides (56), or aptamers (35), to achieve active tumor recognition (35, 51, 56). Instead, HA achieves two roles simultaneously. It can act as a carrier, and as a result of its affinity towards “lock” cell proteins CD44, it takes the role of the targeting ligand, too. However, HA-mediated targeting should not be regarded as universally predictable, especially given the different CD44 isoforms across different cancer types. Additionally, HA molecular weight affects receptor interactions, inflammatory signaling, and tumor biology (46, 48, 68). Therefore, receptor expression alone might be insufficient as a general predictor of the targeting efficiency of HA-based systems, and, for nanoplatform design, the receptor phenotype and molecular characteristics of HA should be considered.

Inconsistent polymer size and degree of deacetylation, as the main limitations of chitosan, arise from differences in chitosan origin, which, in turn, affect polymer properties such as its charge density, solubility, viscosity, degradation, and the potential for biological interactions (44, 55, 57, 70). Under physiological conditions at pH 7.4, the positive charge on native chitosan is neutralized, thereby restricting its solubility (44). Additionally, the positive charge of chitosan may contribute to nonspecific protein adsorption, i.e., protein clumping and rapid elimination from the body triggered by the immune system (51). The inadequate mechanical performance of scaffolds is usually addressed by blending or reinforcing chitosan with other materials (44, 57). Yet, HA faces a different set of challenges, such as changes in biological activity with changes in molecular weight, or in targeting efficiency due to different receptor isoforms (72), The degradation of HA by the enzyme hyaluronidase typically alters nanocarrier stability and therapeutic release (65). Furthermore, the scientists must control the degree of chemical modification of HA, as extensive substitution often alters the polymer's conformation, potentially hindering receptor recognition of HA (72). Thus, the major challenge for chitosan is to standardize an engineering material (44) whereas for HA it is to preserve and predict the behavior of a biologically interactive material (65, 72).

The formation of polyelectrolyte complexes and multilayer structures between chitosan and HA via electrostatic interactions, arising from their opposite charge states, provides a rational basis for hybrid nanocarrier design (51, 70). Combining the effective drug entrapment, structural integrity, pH responsiveness, and intracellular delivery provided by chitosan with CD44-mediated targeting of HA, the chitosan–HA systems have been investigated for the delivery of chemotherapeutics, peptides, RNA, and combined therapeutic cargoes (61, 70). Complementing each other's properties, these multicomponent nanosystems have been shown to be suitable candidates for multimodal anticancer therapy, demonstrating improved performance, such as tumor accumulation, receptor-mediated internalization, sustained or pH-responsive release, and enhanced therapeutic efficacy (51, 61, 70). Hybrid systems that rationally combine the engineering versatility of chitosan with the biological selectivity of HA may therefore represent a particularly promising route toward next-generation bio-based nanophotothermal platforms. Future development of hybrid systems should move beyond a simple combination of two polymers and instead optimize their molecular weight, charge ratio, degree of chemical modification, and spatial organization according to the intended therapeutic mechanism. This structure–function-driven approach could provide simpler, more reproducible, and clinically relevant nanoplatforms in which each biomaterial performs a clearly defined and complementary therapeutic role.

## 5 Translational challenges and future perspectives

Despite substantial progress in bio-based nanophotothermal drug delivery, translation from experimental models to routine clinical practice remains limited and faces serious multidimensional bottlenecks (71). While clinical studies of hyaluronic acid- and chitosan-based nanophotothermal systems have shown remarkable preclinical efficacy, their clinical translation remains limited (85). At present, the most advanced clinical experience with nanoparticle-mediated photothermal therapy comes from inorganic gold nanoshell platforms (e.g., AuroLase®), which exhibited a favorable safety profile with no serious treatment-related adverse events, while 68% of treated lesions showed negative targeted biopsies one year after treatment. Earlier clinical studies confirmed successful focal tumor ablation in 94% of treated patients, preserving genitourinary function (86). These encouraging results demonstrate the clinical potential of photothermal nanomedicine and provide an important translational benchmark for future bio-based nanophotothermal platforms.

Still, a high-priority frontier in precision medicine is the development of “smart” diagnostic and therapeutic systems. The outcomes from preclinical trials can't predict the true performance of drug delivery systems when implemented in clinical trials (47, 73). Clinical translation requires simultaneous control of material composition, nanoparticle architecture, drug loading, biological interactions, irradiation conditions, manufacturing reproducibility, and long-term safety (6, 42, 49). Multifunctionality in the system not only introduces additional components and more synthesis steps but also quality control requirements. Basically, the development of simpler, reproducible, and scalable formulations should be prioritized because clinical evidence indicates that their further progress is greater than that of more complex systems (46, 61).

Process optimization of many fabrication techniques, such as electrospinning, cryogelation, and 3D bioprinting, is achieved at the laboratory scale, but struggles to attain reproducibility and cost-effectiveness in large-scale production. These platforms face regulatory obstacles in achieving complex approval pathways. This is due to a lack of batch-to-batch consistency in particle-size distribution, drug-loading efficiency, and surface functionalization (44, 73). Thus, a strategy for decreasing the gap between laboratory trials and clinical applications for nanocarrier systems is the implementation of industrial-scale Chemistry, Manufacturing, and Controls (CMC) procedures and Good Manufacturing Practice (GMP), supported by rigorous quality control protocols (46, 63, 73). Since biopolymers of different origins may result in different characteristics like molecular weight, molecular weight distribution, and the degree of deacetylation, which, on the other hand, define charge density, solubility, and the kinetics of enzymatic degradation of biopolymers (57), one of the primary requirements for establishing the quality control protocols is the standardization of raw materials of biopolymer (44). Additionally, quality-control procedures for nanomaterials in biomedical applications should include protocols for removal of residual synthesis reagents and impurities (30). Also, successful surface functionalization and colloidal stability of these systems are achieved by standardized physicochemical characterization of nanocarriers, monitoring particle size, polydispersity index (PDI), and zeta potential (52). Furthermore, clear drug-release specifications for stimuli-responsive nanoplatforms would ensure consistency of these “smart” functions across production lots (42). Strong compliance with the international series of standards for biological evaluation, i.e., ISO 10993, provides a necessary regulatory framework to ensure long-term biosafety and hemocompatibility of medical devices (71).

Biological heterogeneity, i.e., therapeutic outcomes in animal models and those in human patients, complicates the preclinical-clinical transition. Namely, standard tumor models used in *in vivo* studies often lack the specificity and biological complexity of human malignancies, particularly regarding the reliability of the EPR effect, which varies among patients (animals or humans) and tumor types. Thus, nanosystems reaching the tumor can encounter physical and biological barriers in the TME that restrict their progress deep into the tumor core (21, 42). As shown for HA-mediated targeting through CD44 recognition, this receptor exists as a standard isoform (CD44s), and depending on tumor subtype, disease stage, and metastatic potential, numerous variant isoforms (CD44v) may be differentially expressed (48, 72). In particular, some CD44v isoforms, due to steric hindrance caused by their larger size or sialylation, have reduced affinity for HA binding compared to CD44s, resulting in inconsistent targeting efficiency across patients, tumor stages, or responses to different anti-tumor treatments (48). For this reason, rather than adopting a one-size-fits-all approach (44), future clinical translation should focus on personalized strategies with quantitative evaluation of CD44 expression and select the patients most likely to benefit from HA-mediated targeting (42).

Regarding the dearth of research on the long-term biodistribution, toxicity, and metabolic clearance of these materials under physiological conditions, the long-term biological fate remains insufficiently characterized. Although many studies have assessed acute cytotoxicity, short-term tumor suppression, body weight, and histology of major organs (35, 50), there is a lack of research on repeated administration, chronic inflammation, immunogenicity, degradation products, reproductive toxicity, and delayed organ accumulation (59). While bio-based polymers may degrade into relatively safe products, some chemical modifications and the hybrid nature of nanophotothermal systems, which contain metal/oxides (7, 22), graphene derivatives (87), semiconducting polymers (10), and others, may lead to undesirable cellular uptake or alter degradation kinetics. Namely, inorganic photothermal cores such as gold nanoparticles or carbon derivatives that are not efficiently cleared by renal filtration are recognized and internalized by macrophages of the mononuclear phagocyte system, which can result in extended retention in vital organs, e.g., the liver and spleen, raising concerns regarding long-term toxicity and chronic inflammation (31, 42). On the other hand, surface coatings of inorganic nanoparticles with HA may prolong circulation but do not eliminate the need for eventual clearance of the inorganic core (73). As a result, future studies related to clinical translation of bio-based nanophotothermal systems should focus on developing biodegradable or metabolizable photothermal agents, along with standardized pharmacokinetic and pharmacodynamic analyses, toxicological studies, and comprehensive assessment of biodistribution, clearance, and potential organ accumulation.

During photothermal treatment, excessive heating can damage healthy tissue, but insufficient or heterogeneous heating usually leaves viable tumor regions and promotes recurrence (59), highlighting the need for standardization and strict control of key irradiation parameters, including fluence, exposure time, irradiation geometry, and spot size (58, 80). Due to the lower scattering inside tissues and thus deeper tissue penetration, higher maximum permissible exposure, lower tissue autofluorescence, and smaller unwanted surface heating, the proposed future clinical standardization protocols of PTT should implement the NIR-II biological window over the conventional NIR-I (61, 77). Because determining the temperature profile in the treated tissue is very important for PTT, photoacoustic imaging and MRI thermometry, which are more appropriate for assessing temperature distribution in deep tissues than infrared thermal imaging, which is better suited for controlling the thermal distribution in superficial tumors (14, 30). Automated real-time regulation of the laser output in response to tissue temperature, which will avoid insufficient or excessive heating resulting in tumor recurrence or healthy tissue damage, respectively, is enabled by the integration of these imaging modalities with closed-loop temperature feedback systems, such as photothermoelectric (PTE) hydrogels (63). Computational modeling may additionally help optimize the standardization of laser irradiation parameters and spatial temperature profiles.

Future progress of these nanoplatforms will depend on a shift from complex microscopic vehicles to functionally integrated, smart, multi-responsive systems with biodegradable architectures that have defined degradation pathways, preventing premature release but enabling sequential drug release while remaining clinically manageable. While triple-responsive systems (e.g., pH/GSH/thermal) have demonstrated highly selective, sequential drug release and thus excellent efficacy in preclinical animal models (59), their clinical implementation is hindered due to manufacturing barriers induced by their high structural complexity, increased number of production steps, raised production costs, and reduced batch-to-batch reproducibility (33, 73). As the regulatory agencies require comprehensive data on CMC parameters and long-term safety for these complex “combination products”, such multifunctional platforms also face a heavier regulatory burden, additionally restricting their clinical transition (61). Consequently, the improved therapeutic efficacy of highly sophisticated nanoplatforms should be carefully balanced against their manufacturability and clinical feasibility. Following the scientific interest in higher-order responsive systems, future clinical translation is likely to prioritize simpler dual-responsive formulations that offer an optimal balance between “smart” functionality, manufacturing scalability, regulatory compliance, and cost-effective clinical implementation.

The development of such NPTSs, in which image-guided thermal control and rationally selected therapeutic combinations are provided, can be supported by Artificial Intelligence and computational modeling. To make these novel methods very useful, one needs to provide standardized, high-quality experimental datasets on which the computer models can be trained and improved. It means that a reproducible system in which a balance among targeting, controlled release, photothermal efficacy, multimodal synergy, biodegradation, safety, and manufacturability is established could be developed only through early-stage collaboration among materials scientists, pharmaceutical technologists, oncologists, imaging specialists, toxicologists, manufacturers, and regulatory authorities.

## 6 Conclusions

The application of bio-based nanophotothermal drug delivery systems, which integrate multiple functions such as targeted drug delivery, photothermal therapy, and stimuli-responsive release, in addition to multimodal therapeutic approaches, is a strategy for overcoming the limitations of conventional cancer therapy. This review analyzes two of the most versatile natural polysaccharides, chitosan and hyaluronic acid, which have complementary physicochemical and biological properties and are widely used in medicine and pharmacy. Their complementary characteristics make these bipolymers suitable for preparing multifunctional nanoplatforms with improved therapeutic performance. While chitosan serves as an engineering matrix with strong structural adaptability, pH-stimulus responsiveness, and hydrogel-forming capability, hyaluronic acid enables precise drug delivery. Issues related to the biological complexity of the tumor microenvironment, manufacturing reproducibility, scalability, long-term biosafety, pharmacokinetics, and regulatory approval remain the main constraints for successful bench-to-bedside translation. Proposed solutions to address these and similar problems include the introduction of standardized protocols for material characterization, the use of relevant animal models, and robust quality control. Additionally, closer collaboration among experts across branches such as materials science, nanotechnology, and oncology, as well as regulatory bodies, would help resolve this issue.

This work provides a comprehensive understanding of chitosan and hyaluronic acid, presenting their capability in multifunctional systems suitable for theranostic therapies of different cancer types. Yet, this study has several limitations. As a narrative review, it focuses primarily on recent advances involving only these two biopolymers (chitosan and hyaluronic acid) and therefore does not comprehensively evaluate all bio-based nanomaterials or quantitatively compare therapeutic outcomes across studies. Future work would expand the analysis to additional bio-based platforms and explore clinically translatable hybrid systems that integrate targeted delivery, theranostic monitoring, and adaptive multimodal therapy.
